# A second locus associated with high-level resistance to Bt Cry1Ac in *Trichoplusia ni*

**DOI:** 10.1128/aem.02329-25

**Published:** 2026-04-14

**Authors:** Xiaowei Yang, Rey O. Cotto-Rivera, Zhangjun Fei, Ping Wang

**Affiliations:** 1 Department of Entomology, Cornell University 116317, Geneva, New York, USA; 2 Boyce Thompson Institute 53401, Ithaca, New York, USA; Universite de la Reunion, Ste Clotilde, France

**Keywords:** *Bacillus thuringiensis*, Bt resistance, Cry1Ac, resistance gene mapping, resistance dominance

## Abstract

Previous studies on the toxicity of the insecticidal protein Cry1Ac from *Bacillus thuringiensis* (Bt) have indicated the presence of multiple pathways of Cry1Ac toxicity in the cabbage looper, *Trichoplusia ni*. Consistently, the high-level resistance to Cry1Ac evolved in greenhouse populations of *T. ni* has been identified to be associated with an *ABCC2* gene mutation and an additional yet-to-be-identified gene mutation. In this study, the second resistant trait to Cry1Ac in *T. ni*, Cry1Ac-R2, was genetically mapped by biphasic linkage analysis, using bulked segregant analysis by whole-genome sequencing (BSA-seq) and amplicon sequencing (Amp-seq). The Cry1Ac-R2 was localized in a 675-kb region on chromosome 9. Of the 37 genes in the Cry1Ac-R2 locus, 25 were expressed in the midgut of *T. ni* larvae. Among those midgut genes are a cluster of aminopeptidase N (APN) genes, including *APN1* and *APN6*. Analysis of the dominance of Cry1Ac-R2 revealed that the dominance at a high dose of Cry1Ac could be significantly influenced by the genetic background of *T. ni* strains. The results from this study demonstrated that resistance to Cry1Ac in an insect may involve multiple gene mutations with various types of dominance, and the dominance can be influenced by the genetic background of an insect population.

**IMPORTANCE:**

The soil bacterium *Bacillus thuringiensis* (Bt) and its insecticidal Cry proteins are widely used for insect control in agriculture and public health. Continuing success of Bt biotechnology requires a better understanding of the insect genes involved in the toxicity of Bt proteins and their mutations leading to Bt resistance. Cry1Ac is a major Bt protein used for *Lepidoptera* pest control, and resistance to Cry1Ac has occurred in field populations of insects, including the cabbage looper, *Trichoplusia ni*. The results from this study confirmed that Cry1Ac resistance in an insect may involve multiple gene mutations and both dominant and recessive resistant traits. This study also revealed that the dominance of a resistance trait can be influenced by the genetic background of an insect population, which could complicate research data interpretation and may affect the efficacy of Bt toxins for control of different insect populations in the field.

## INTRODUCTION

*Bacillus thuringiensis* (Bt) is a ubiquitous soil bacterium distributed worldwide and an important insect pathogen that has long been used as a major bioinsecticide for insect pest control in agriculture and public health (1–3). Bt strains produce various virulence factors that contribute to their pathogenicity and toxicity in insects. The insecticidal Cry proteins are major virulence factors in parasporal crystals produced during sporulation of the bacterium, and a number of Cry toxins have been widely expressed in transgenic crops (Bt-crops) in agriculture to confer insect resistance (4, 5). However, the widespread applications of Bt sprays and transgenic Bt-crops exert an intense selection pressure on insect populations in the field, which has led to evolution of insect resistance to Bt toxins and continues to threaten the long-term efficacy of Bt biotechnology in agriculture (6). To sustain the success of Bt biotechnology for insect pest control, it is essential to better understand the insect genes involved in the toxicity of Bt proteins and their mutations leading to Bt resistance.

The pathogenesis of Cry toxins in insects is complex, and mechanisms of Bt resistance may vary depending on the toxins and the target insects (7–10). Insect strains resistant to Cry proteins are valuable research systems for understanding the modes of action of Bt proteins and the gene mutations to confer resistance. Cry1Ac is a major Cry protein used for lepidopteran pest control, and resistance to Cry1Ac has occurred in field insect populations (11–13). High-level resistance to Cry1Ac in insects commonly shows as “mode 1 type” resistance (14) resulting from disruption of the specific toxin binding to midgut receptors. Insect midgut proteins that have been associated with the toxicity of Cry1Ac as receptors include ABC transporters in subfamily C, a cadherin protein, aminopeptidases N (APNs), and the membrane-bound alkaline phosphatase (8, 15–17). The binding of Cry1Ac to midgut receptors also involves carbohydrates associated with the proteins (18–22). The ABC transporter ABCC2 in insects has been experimentally shown to serve as a functional receptor for Cry1Ac by overexpression of a *Lepidoptera ABCC2* in cell culture (16) and in transgenic *Drosophila* larvae (23), and experimental knockout of *ABCC2* in *Lepidoptera* larvae has been shown to reduce larval susceptibility to Cry1Ac (24–26). In several Cry1Ac-resistant insects, the high-level resistance is associated with mutations in the *ABCC2* gene (25–28). However, knockout mutations in the *ABCC2* gene in insects often only lead to a low-to-moderate level of resistance (25, 26, 29). These studies have indicated that there exist multiple pathways of Cry1Ac toxicity in insects. Therefore, a high level of resistance often requires multiple gene mutations to disrupt the multi-pathways of Cry1Ac toxicity (30–32). In Cry1Ac-resistant *Heliothis virescens* and *Plutella xylostella*, an *ABCC2* mutation together with a disruptive mutation in the midgut *cadherin* or in *ABCC3* has indeed been identified to contribute to high-level resistance to Cry1Ac (25, 27).

The cabbage looper, *Trichoplusia ni*, developed resistance to Bt sprays in greenhouses (33). The high-level resistance to Cry1Ac is conferred by a major disruptive 4-bp insertion mutation in *ABCC2* (resistance trait Cry1Ac-R1) and an additional mutation to be identified (resistance trait Cry1Ac-R2) (26, 34). Cry1Ac-R1 and Cry1Ac-R2 have been isolated into individual *T. ni* strains, and their resistant phenotypes have been separately determined. Cry1Ac-R1 without Cry1Ac-R2 confers a moderate level of resistance to Cry1Ac (resistance ratio ~90-fold), and Cry1Ac-R2 alone confers a minimal level of resistance (resistance ratio ~3.5-fold) (34). Further studies have confirmed that neither the midgut cadherin nor the ABCC3 genes are involved in the high-level resistance to Cry1Ac in *T. ni* derived from the greenhouse (26, 32). Our recent study further indicated that TnCry1Ac-R2 is an incompletely dominant trait and is genetically independent of the *ABCC2* gene (34). In this study, genetic mapping of TnCry1Ac-R2 was conducted by biphasic linkage analysis using bulked segregant analysis by whole-genome sequencing (BSA-seq) and amplicon sequencing (Amp-seq), and the midgut-expressed genes in the mapped resistance locus were identified, and their levels of expression were determined. In addition, differential dominance of TnCry1Ac-R2 under different genetic backgrounds of *T. ni* strains was revealed and analyzed.

## MATERIALS AND METHODS

### *T. ni* strains and Bt toxin Cry1Ac

The *T. ni* strains used in this study were as follows: i) the TnCry1Ac-R strain (35) originated from a Bt-resistant greenhouse population (12), ii) the susceptible laboratory Cornell strain (12), iii) the susceptible *T. ni* strain purchased from Benzon Research (Carlisle, PA) with a different genetic background from the Cornell strain, and iv) the *ABCC2* knockout mutant strain ABCC2E4-3 generated from the Cornell strain by CRISPR/Cas9 mutagenesis (26). Bt Cry1Ac protoxin was prepared from the *B. thuringiensis* strain HD-73, as previously reported (12).

### Identification of the genetic linkage group of Cry1Ac-R2 in *T. ni*

**Fig 1:**
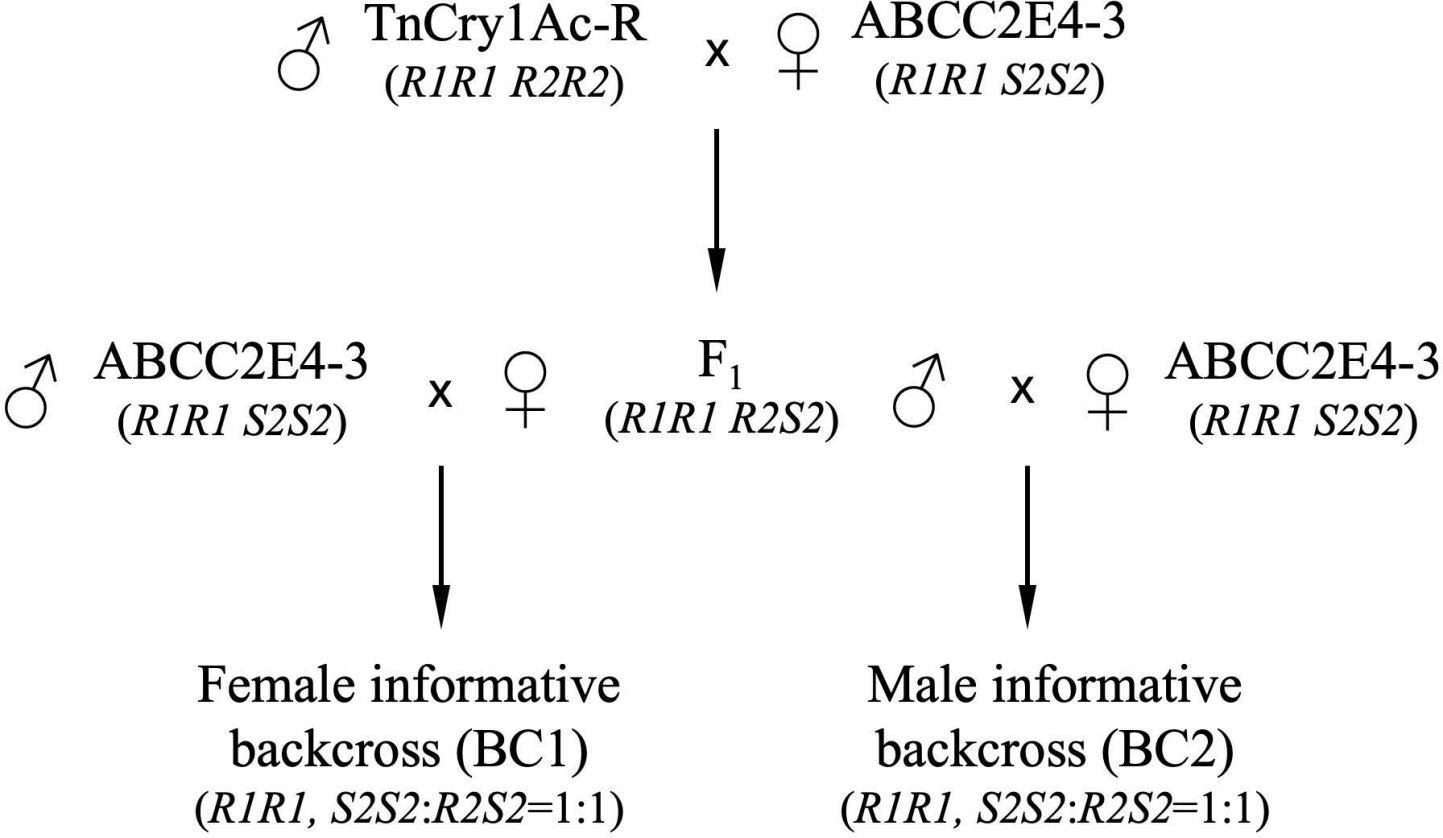
Generation of male informative and female informative backcross populations for genetic linkage analysis of Cry1Ac-R2 in *T. ni*. F1 individuals (genotype: *R1R1 R2S2*) were prepared by crossing a TnCry1Ac-R male (*R1R1 R2R2*) with an ABCC2E4-3 female (*R1R1 S2S2*). Female informative backcross (BC1) and male informative backcross (BC2) populations were generated for biphasic linkage mapping of Cry1Ac-R2. The BC1 individuals with and without Cry1Ac selection were used to determine the linkage group of Cry1Ac-R2. The BC2 individuals with and without Cry1Ac selection were used to map Cry1Ac-R2 within the resistance linkage group.

The biphasic linkage mapping method for lepidopterans (36) was used to map the Cry1Ac-R2 locus. The resistant allele of the Cry1Ac-R2 gene is designated *Cry1Ac-R2* (*R2*), and the susceptible allele is designated *Cry1Ac-S2* (*S2*). As the resistant trait Cry1Ac-R2 is incompletely dominant (26, 34), a female informative backcross family of *T. ni* in the *ABCC2-*knockout mutation background (*ABCC2^−^ABCC2*^−^, or *R1R1*) was prepared for the genetic association analysis to identify the linkage group of *Cry1Ac-R2* (Fig. 1). Specifically, the TnCry1Ac-R strain was crossed with the *T. ni* ABCC2E4-3 strain to generate their F_1_ family, and females from the F_1_ family were backcrossed with males from the ABCC2E4-3 strain to obtain the female informative backcross population (named as BC1). All individuals in BC1 had a frameshift knockout mutation in both copies of the *ABCC2* gene (*R1R1*)—the 4-bp insertion in *ABCC2* from the TnCry1Ac-R strain and the 68-bp deletion in *ABCC2* from the ABCC2E4-3 strain (26). For the Cry1Ac-R2 gene, 50% of the BC1 individuals were expected to be homozygous of the susceptible allele *Cry1Ac-S2* (*S2S2*) and the other 50% to be heterozygous of the susceptible and resistant alleles of *Cry1Ac-S2* and *Cry1Ac-R2* (*S2R2*), respectively. Neonate larvae of the BC1 population were selected on diet spread with Cry1Ac at 2 µg/cm^2,^ which is approximately five times of the LC_50_ of Cry1Ac in the ABCC2E4-3 strain (*R1R1 S2S2*) and about the LC_50_ in F_1_ progenies (*R1R1 S2R2*) from ABCC2E4-3 × TnCry1Ac-R. Under this Cry1Ac dose, the Cry1Ac-R2 trait is incompletely dominant (26). The larvae were reared on the diet with Cry1Ac, and those that had reached the fifth instar were collected as the more resistant heterozygous *Cry1Ac-R2/Cry1Ac-S2* genotype (*R1R1 S2R2*) over the relatively susceptible homozygous *Cry1Ac-S2* genotype in the *ABCC2* knockout background (*R1R1 S2S2*). A group of larvae from the same BC1 population were also reared on a diet without Cry1Ac treatment and used as nonselected controls. These collected larvae were used for BSA-seq to analyze the segregating backcross population BC1 with and without Cry1Ac selection to identify the genetic linkage group associated with the Cry1Ac-resistant Cry1Ac-R2 trait.

For BSA-seq, genomic DNA was prepared from a pool of 160 Cry1Ac-selected BC1 larvae and a pool of 86 non-Cry1Ac-selected BC1 larvae using the E.Z.N.A. Tissue DNA Kit from Omega Bio-Tek (Norcross, GA). The DNA samples were fragmented to 600 bp to construct sequencing libraries using the Illumina TruSeq Nano library preparation kit and sequenced on an Illumina NextSeq500 platform (150-bp paired-end sequencing) at the Genomics Facility of Cornell University (Ithaca, NY). The raw Illumina sequence reads were processed using Trimmomatic (37) to remove adapters and low-quality sequences, and were then mapped to the genome assembly of the *T. ni* Cornell strain (38). Single-nucleotide polymorphisms (SNPs) from the Cry1Ac-selected and non-Cry1Ac-selected BC1 populations were identified using the software package GATK (39), and allele frequencies of the SNP sites were calculated as previously reported (40). To analyze the selection of Cry1Ac resistance-associated SNPs in larvae by Cry1Ac feeding, kernel density estimate (KDE) plots were generated (41) to visualize the changes in the abundance distribution of allele frequencies in each chromosome in response to the selection with Cry1Ac.

### Fine mapping of the *Cry1Ac-R2* locus on chromosome 9 in *T. ni*

For fine mapping the gene *Cry1Ac-R2* on chromosome 9, a male informative backcross population (named as BC2) was generated by crossing the TnCry1Ac-R strain with the ABCC2E4-3 strain to obtain F_1_ progeny and then crossing F_1_ males with females from ABCC2E4-3 (Fig. 1). BC2 larvae were selected on a diet with Cry1Ac at 2 µg/cm^2,^ as described above. A group of BC2 larvae reared on a diet without Cry1Ac was used as non-Cry1Ac-selected controls. SNP/INDEL mapping markers on chromosome 9 were selected from variants identified in the TnCry1Ac-R genome resequencing data set (26), and 48 PCR primer pairs were designed to amplify the marker fragments for Amp-Seq (Table 1), as we previously described (40). *T. ni* genomic DNA samples were prepared from a pool of 253 Cry1Ac-selected BC2 larvae and a pool of 257 non-Cry1Ac-selected BC2 larvae, respectively, using the E.Z.N.A. Tissue DNA Kit. PCR amplicons from the DNA samples were generated by PCR with the 48 PCR primer sets, using the iProof High-Fidelity PCR Kit (Bio-Rad, Hercules, CA, USA), and the amplicons from the same treatment group were pooled. Sequencing libraries of the amplicons were prepared using the Illumina TruSeq Nano library preparation kit and sequenced on an Illumina NextSeq500 platform (150-bp paired-end) at the Genomics Facility of Cornell University.

**TABLE 1:** Primers used for PCR amplification of amplicons for Amp-seq

Primer set		Sequence	Position on chromosome 9	PCR product (bp)
1	Forward	GTATTCAGAGTAGGGTGTGAATGT	1,270,927	544
	Reverse	TGTATGTCTAGCAGGTGGATTTG	1,271,471	
2	Forward	CCGCTACTGAACACTATCGTA	1,528,061	661
	Reverse	CGACGGCCTTATTGCTAGAT	1,528,722	
3	Forward	CCTGTTGAACCGCTAGACATTAG	1,767,405	584
	Reverse	GTAAGCTTCGCACAGTTAGGA	1,767,989	
4	Forward	GCTACATATCACTCATCGCTAC	1,867,402	547
	Reverse	CGATTTGATTCTCGGGATCTAGG	1,867,949	
5	Forward	GTTGCCACAGCAGAATACATAG	1,975,571	562
	Reverse	GCTTATACACAGACGCCTTATC	1,976,133	
6	Forward	GGTACCAAAGTTACGAGTAGTAG	2,024,328	378
	Reverse	TGCATAAACAATGCACACTT	2,024,706	
7	Forward	GAAGCTGCAACATTGGATGG	2,073,992	433
	Reverse	CTTACCAGTTTGCTGTTTGTTGA	2,074,425	
8	Forward	GATAACGGTGTTCCGTGAGT	2,094,322	353
	Reverse	CCTTTCCTCATAGCTGTCATGT	2,094,675	
9	Forward	GGGAAGCTGTACGCTACAT	2,162,567	377
	Reverse	CCAGGAATGGACGTTTGGTATG	2,162,944	
10	Forward	GAAGCCATTGTTGACGGGAT	2,253,615	362
	Reverse	GAGATTTGGGAGACGGATGG	2,253,977	
11	Forward	CAGAATAAGCATTCATGTGTTAC	2,342,930	572
	Reverse	GTTATACCCGCGACTTCGTTAG	2,343,502	
12	Forward	GGTTCTGAACACACCCTGA	2,442,248	389
	Reverse	GCTGACAGGTTCTTAACGATAG	2,442,637	
13	Forward	GTAAGAGTGGTGAGGGCAATAG	2,546,746	714
	Reverse	TGGCTCGGAACTGCTAAAC	2,547,460	
14	Forward	CTTGTACGACGCAGTTTGTAT	2,636,392	496
	Reverse	GACTGGCGTATGATCGAATCT	2,636,888	
15	Forward	CTATGCACATTGGGCGAAAC	2,721,717	302
	Reverse	GATGGTGATATGTGAGGTGTAG	2,722,019	
16	Forward	CAGGATGGGCTTAGACATAACA	2,813,373	356
	Reverse	CAGCTCGCACAGATGACT	2,813,729	
17	Forward	GCCTCTAATTGCGAACGTTATG	2,911,624	503
	Reverse	TGCCTCAATATGCGGGAA	2,912,127	
18	Forward	CATAATGGCGGGCATCTCA	3,327,419	478
	Reverse	GAGTCTGGACCAACATTCAAAGA	3,327,897	
19	Forward	CACTTCGTTTCGTGAGTTCTTC	3,667,757	345
	Reverse	GGTCGTTGTTTCTTACTTATTC	3,668,102	
20	Forward	GATGGAACTGGCCACTCTG	3,782,249	749
	Reverse	CCAGTGTTGGTCAAGTGAGT	3,782,998	
21	Forward	GCCAAAGTTCAGATACTCGTTC	4,635,304	395
	Reverse	GGTACTTAGGCTTATTTGTAAGGA	4,635,699	
22	Forward	CTTCTCATATCTAGCCAGCCCT	4,731,012	694
	Reverse	GTTTAATCGTTTATGCAGCTGGT	4,731,706	
23	Forward	CTGGTTCCGCCCATTGTATT	4,826,566	356
	Reverse	CTTTGGTTTACAAGGGCGTAAG	4,826,922	
24	Forward	CCATATTGTACGAGTCAGGGTAAG	4,953,916	295
	Reverse	CATCATAGAGATACTGTGGCTATC	4,954,211	
25	Forward	CTGTTTGGTTGGCGACATTC	5,062,280	273
	Reverse	GTTACTGTACGTTAACGAGATG	5,062,553	
26	Forward	GGTCGACAACCCTAATAGATACTG	5,154,509	514
	Reverse	GCATCCTCTCGCCTGAAGAA	5,155,023	
27	Forward	GATTCTGGACGGTGGCAAG	5,255,852	504
	Reverse	GTTACGTGGTCAAAGTTTACA	5,256,356	
28	Forward	ACTGATCTATACCTATCAGTCGT	6,649,406	323
	Reverse	CCTACCGATGGTGAAAGAACT	6,649,729	
29	Forward	GTGTAGTCTTCAAAGCACATTCC	6,762,647	565
	Reverse	CACTTCCTTATTCACATTTCGG	6,763,212	
30	Forward	CGATTGCAACAAGGTTTGTTAAG	6,864,555	390
	Reverse	CATTCAACTAGACTTCTAAGGCA	6,864,945	
31	Forward	CCCGCTAACAGCATAGTGAA	9,011,689	471
	Reverse	CCACTTAAGTTCTAGATGGTGT	9,012,160	
32	Forward	CTGTGTACTGGTGACCTAAATTC	9,981,535	267
	Reverse	CAATCTTGGACTGCGGACAAT	9,981,802	
33	Forward	CTCACGAACTTTGGTAGACAGG	10,087,559	614
	Reverse	GGGACACAAAGAGATGGTGT	10,088,173	
34	Forward	CCTAGCCTAATCCGGTAAGAC	10,189,242	560
	Reverse	GAGTCAATCTCTGCAGGATACC	10,189,802	
35	Forward	TGTTCGCGCTTATCAATTTCTG	10,287,749	440
	Reverse	CCATCCAAAGCACACAGATTTC	10,288,189	
36	Forward	GAGGGCACTGTACGAATCAC	10,398,424	677
	Reverse	CCACAGTTATTTCCCTCTCACA	10,399,101	
37	Forward	CAGAACTCGTCAGCCAAAGT	10,511,091	396
	Reverse	CGGAATCCGTATCCCACATAG	10,511,487	
38	Forward	CTTGGTCGTGATCCCTGTCTA	10,622,622	556
	Reverse	CGTAAACTGCCACAGATACCTA	10,623,178	
39	Forward	CGAGGGATACAGCACATTTGT	10,734,239	401
	Reverse	TGTGGCCAATAGCAAACAAG	10,734,640	
40	Forward	AGCTCTGCTCCTTGTTCTTC	10,839,102	576
	Reverse	CCCGTCAAAGGTAGAGTTATGAG	10,839,678	
41	Forward	ACAACCCTAAACCGCTATGG	10,917,949	516
	Reverse	CGGAATCCTCAGACTCAAGA	10,918,465	
42	Forward	CCTGAGTCAGCTCCTAGATTTC	11,065,021	382
	Reverse	GTTTCGTCCCGACTTGTGATAG	11,065,403	
43	Forward	TCCAAACATAAAGTAGGGCAGT	11,229,448	395
	Reverse	CCGTCTGTTCAATTTCTGTTTGAG	11,229,843	
44	Forward	CGGAACAAGATCGCGAAACA	11,329,127	649
	Reverse	CCAACATCGCGTCAACATAGT	11,329,776	
45	Forward	CGTGCATAAACGCTTCAACAA	11,429,199	411
	Reverse	CTTACCAGTGGCTTCGAGATG	11,429,610	
46	Forward	ACCTTGTGTCTGGTGTGAAG	11,498,404	615
	Reverse	CCAAGCCTAGTAACCTGTCAAT	11,499,019	
47	Forward	CACTCCTAAAGAAGCGGAAATC	11,584,169	398
	Reverse	CGTGAAACGTGTTAAGTGACTG	11,584,567	
48	Forward	TAGTCACGCAACAACATACC	12,015,084	676
	Reverse	GACGAGTAATGTCTTCGCTTCA	12,015,760	

The raw Illumina sequence reads from Amp-Seq were processed using Trimmomatic (37) to remove the adapters and low-quality sequences. The cleaned sequence reads were mapped to the *T. ni* reference genome (38) using BWA (42). SNPs were called using SAMtools (43), omitting the “mark duplicate” step. The allele frequency (AF) of each SNP was calculated in the sequencing populations, using VCFtools (44). As the frequency of the resistant *Cry1Ac-R2* allele in the mapping population BC2 without Cry1Ac selection was expected to be 0.25, the amplicons showing AFs of SNPs in the non-Cry1Ac-selected control population to be >0.5 or <0.125 were considered having nonspecific PCR amplifications and excluded from the data set for mapping the *Cry1Ac-R2* gene.

### Identification of genes in the *Cry1Ac-R2* locus and analysis of the expression of those genes in the larval midgut

The genes in the mapped *Cry1Ac-R2* locus were identified based on the annotation of the *T. ni* genome from the Cornell strain (http://www.tnibase.org) (38). The expression levels of those genes in the larval midgut were analyzed using the RNA-seq data from the fifth instar larval midgut samples of the Cornell strain and the TnCry1Ac-R strain (38). Briefly, the Illumina reads from the RNA-seq of midgut samples were mapped to the *T. ni* genome using HISAT (45), and the gene expression levels were calculated from the counts of aligned RNA-seq reads and normalized to reads per kilobase of transcript per million mapped reads (RPKM).

### Dominance of Cry1Ac-R1 and Cry1Ac-R2 in *T. ni* larvae in response to high-dose Cry1Ac under different genetic backgrounds

The dominance of Cry1Ac-R1 (conferred by *ABCC2* mutation) and Cry1Ac-R2 (associated with the *APN1* locus identified in this study) to a high dose of Cry1Ac in *T. ni* larvae was analyzed using the Cornell strain and the Benzon strain as two susceptible strains with different genetic backgrounds to assess the dominance of Cry1Ac-R2 under the different genetic backgrounds from the two strains. *T. ni* backcross families were generated by crossing the TnCry1Ac-R strain with a susceptible strain (Cornell strain or Benzon strain) and then backcrossing the progeny F_1_ with TnCry1Ac-R, as we previously described (46). Neonate larvae of the backcross families were selected on an artificial diet spread with 0.2 mL of 250 µg/mL Cry1Ac on the surface (7 µg Cry1Ac/cm^2^) in 30 mL rearing cups (35). Larvae from the same backcross families reared on a diet without Cry1Ac were used as non-Cry1Ac selection controls. After 7 days of rearing, the larvae that had developed to ≥2^nd^ instar were collected. To examine the dominance of the Cry1Ac resistance traits associated with *ABCC2* and *APN1*, the genotypes of *ABCC2* and *APN1* in those *T. ni* individuals were determined by diagnostic PCR analyses to differentiate the alleles from the susceptible and resistant strains. For the diagnostic PCR analysis of *ABCC2* alleles, a primer pair 5′-TACCAGCTTTTTAGTAGTCGTTAG-3′ and 5′-GCCCAAGATAGTCTGAAAGTAA-3′ was used to amplify a PCR fragment of 120 bp from the allele of the susceptible strains, but a fragment of 102 bp from the TnCry1Ac-R strain (47). A set of four primers (5′-GGCCTTCCCTTGCTACGATG-3′, 5′-GGGCTTCTCTGTATGTCAAAAGA-3′, 5′-TTGCTACTGGCACCGACCTC-3′, and 5′-GGGTCATGATATTTTATAATTAGCGTTA-3′ ) was included in the diagnostic PCR to differentiate the *APN1* alleles from the TnCry1Ac-R (PCR fragment = 327 bp) and the susceptible *T. ni* strains (PCR fragment = 234 bp). *T. ni* larval DNA preparation and the diagnostic PCR analyses were conducted as previously described (47).

## RESULTS

### Identification of the linkage group associated with the Cry1Ac-R2 trait by BSA-seq

**Fig 2:**
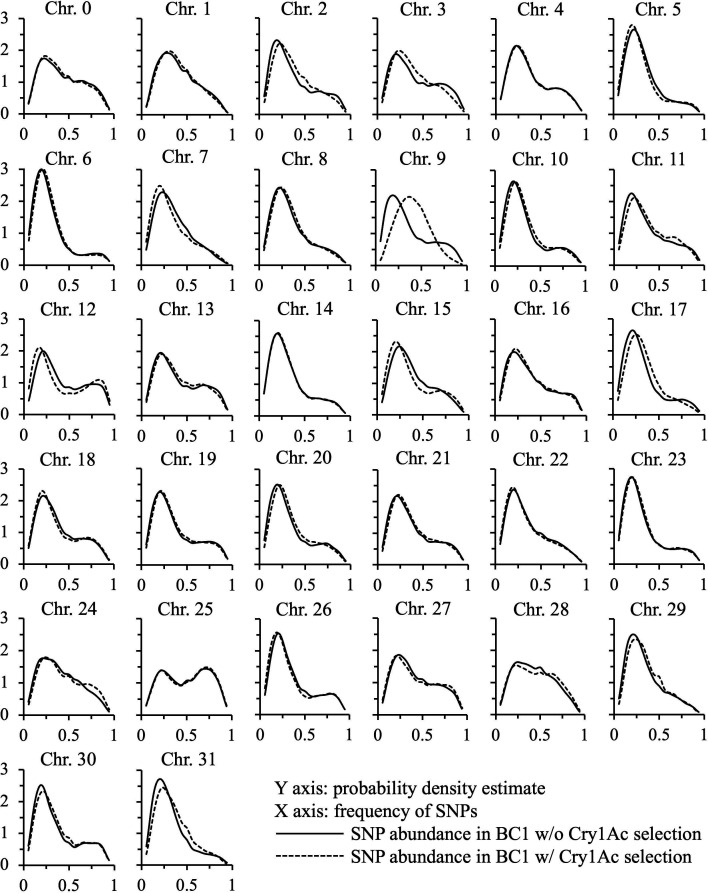
KDE plots showing the abundance of SNP frequencies in each chromosome from larvae of the BC1 population with and without Cry1Ac selection.

BSA-seq of the female informative BC1 mapping populations with and without Cry1Ac selection identified 1,048,575 SNPs. After removal of the SNPs with frequencies < 0.05 or > 0.95, 798,785 SNPs remained and were used to analyze the distributions of the frequencies of these SNPs in each chromosome displayed in KDE plots (Fig. 2). The highest density of SNP frequencies in most chromosomes was between 0.2 and 0.3, which is consistent with the expected frequency of TnCry1Ac-R strain-derived alleles at 0.25 in the BC1 population (Fig. 2). The SNP frequency distributions in 30 of the 31 *T. ni* chromosomes and also in the virtual chromosome 0 in the mapping population BC1 with and without Cry1Ac selection were nearly identical, but a significant shift in the SNP frequency distribution was shown in chromosome 9 (Fig. 2). In chromosome 9, the density peak of the distribution of SNP frequencies was shifted from 0.2 in the nonselected BC1 population to 0.43 after selection of the BC1 population with Cry1Ac, showing selection of the SNPs in chromosome 9 by Cry1Ac. This shift of the most abundant SNP frequency from 0.2 to 0.4 was close to the predicted frequency of 0.25 for the resistance-associated SNPs in BC1 before selection and the frequency of 0.5 in BC1 after selection with Cry1Ac, indicating the association of the Cry1Ac-R2 trait with chromosome 9.

### Mapping of the *Cry1Ac-R2* gene locus on chromosome 9

**Fig 3:**
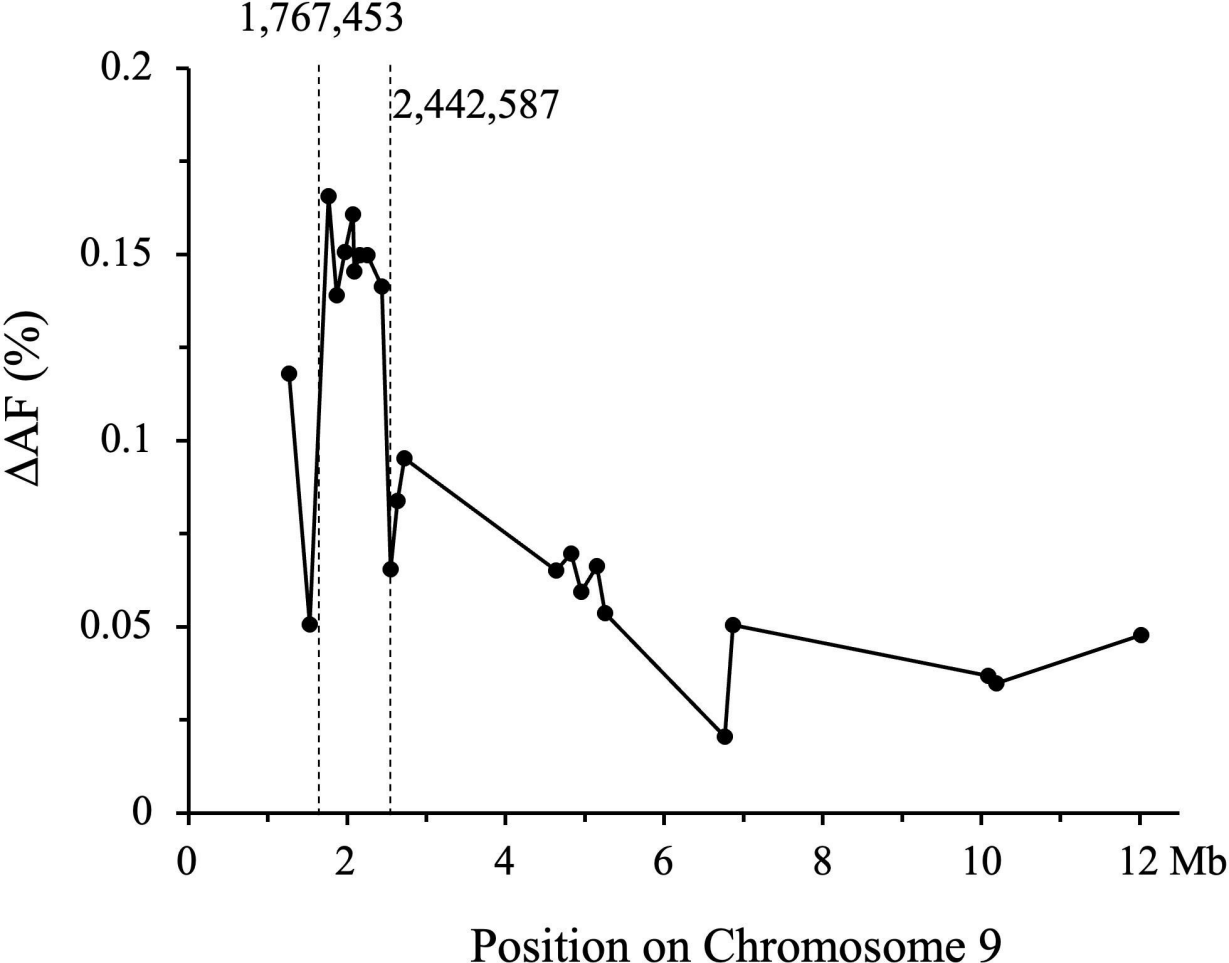
Change of allele frequencies (ΔAF) in amplicons from chromosome 9 of BC2 larvae after selection with Cry1Ac, in comparison with control BC2 larvae without Cry1Ac selection. ΔΑF = AF from BC2 larvae selected with Cry1Ac – AF from BC2 larvae without Cry1Ac selection.

PCR amplicons from 48 PCRs (Table 1) from the male informative backcross family (BC2) with and without Cry1Ac selection were Illumina-sequenced, and the AFs were calculated after the sequencing reads were aligned to the *T. ni* genome. In addition to the removal of the amplicons with AFs in the non-Cry1Ac-selected control population to be <0.125 or >0.5, amplicons that had low sequence reads (<500) were considered unreliable and were also excluded from analysis. The remaining amplicons had an average of 37,755 and 40,466 reads per amplicon for the nonselected and Cry1Ac-selected groups, respectively. The SNPs that showed significantly increased frequencies by selection with Cry1Ac were identified in amplicons localized in a 675-kb locus from position 1,767,453 to 2,442,587 on chromosome 9 (Fig. 3).

### Genes in the Cry1Ac-R2 locus

**Fig 4:**
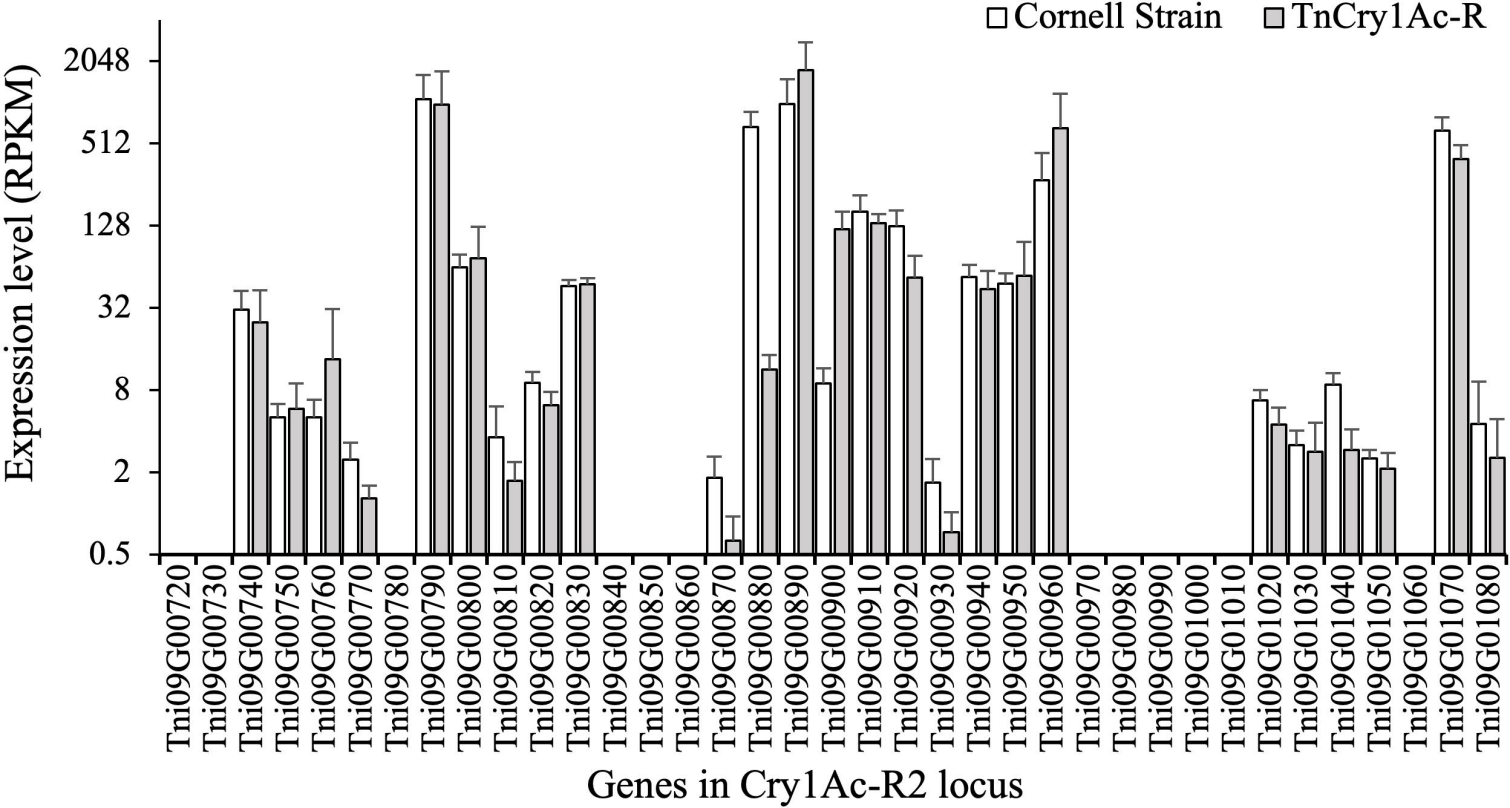
Expression levels of 37 genes in the Cry1Ac-R2 locus in the midgut of fifth instar *T. ni* larvae. The normalized expression levels RPKM of the genes were means calculated from RNA-seq data from six biological replicates of each *T. ni* strains. Error bars represent 95% confidence intervals of the means.

A total of 37 genes were annotated in the 675-kb Cry1Ac-R2 locus in the *T. ni* genome (38) (Fig. 4). From the midgut RNA-seq data with the cutoff of RPKM = 0.5, the expression of 25 genes was detected in the larval midgut, with the RPKM values ranging from 0.5 to 1,360 (Fig. 4). The genes Tni09G00880 (*APN1*, AY836579) and Tni09G00900 (*APN6*, JF303658) showed significantly different expression levels between the Cornell and TnCry1Ac-R strains. The expression level of the *APN1* gene in the midgut of TnCry1Ac-R larvae was 1.6% of that of the Cornell strain (*P* < 0.001 by *t*-test, *n* = 6 for each strain), and the expression level of the *APN6* gene in the TnCry1Ac-R larvae was 1,324.9% of that in the Cornell strain (*P* < 0.001 by *t*-test, *n* = 6 for each strain). For the other genes, the differences in expression between the two strains were no more than 3-fold or were not statistically significant (*P* > 0.01) (Fig. 4).

### Dominance of Cry1Ac-R1 and Cry1Ac-R2 in *T. ni* larvae with different genetic backgrounds under a high dose of Cry1Ac

To examine the dominance of *ABCC2-*associated Cry1Ac-R1 and *APN1* locus*-*associated Cry1Ac-R2, *T. ni* larvae from the backcross family generated from the TnCry1Ac-R with the susceptible Cornell strain and with the Benzon strain were fed on a diet spread with 250 µg/mL Cry1Ac (7 µg/cm^2^) for 7 days. The survivors from the Cry1Ac selection were genotyped for the *ABCC2* and the *APN1* genes as the TnCry1Ac-R1 and TnCry1Ac-R2 markers, respectively. The known resistant *ABCC2* allele (26) is named *ABCC2^R,^* and the susceptible allele is named *ABCC2^S^* (26), whereas the *APN1* allele from the TnCry1Ac-R strain is named *APN1^G^* (46), and the allele from the susceptible Cornell strain or the Benzon strain is named *APN1^S^*. Genotyping of 26 non-Cry1Ac-selected control individuals from the backcross family generated from the cross of TnCry1Ac-R with the Cornell strain showed that 13 individuals were homozygous *ABCC2^R^ABCC2^R^* and the other 13 individuals were heterozygous *ABCC2^R^ABCC2^S^* for the *ABCC2* alleles (homozygotes : heterozygotes = 1:1) (Table 2). However, genotyping 33 survivors from the Cry1Ac selection showed that all the 33 individuals were homozygous for the *ABCC2^R^* allele (homozygotes : heterozygotes = 33:0) (Table 2). This result is consistent with our previous finding that Cry1Ac resistance associated with *ABCC2^R^* is a recessive trait in the backcross family generated with the *T. ni* Cornell strain (26). Similarly, genotyping of the *ABCC2* gene in 51 Cry1Ac-selected individuals of the backcross family generated by crossing the TnCry1Ac-R with the susceptible Benzon strain also showed that all 51 individuals were homozygous for the resistant *ABCC2^R^* alleles (homozygotes : heterozygotes = 51:0) (Table 2), consistent with the recessive Cry1Ac resistance trait conferred by the *ABCC2* gene in the backcross family generated with the *T. ni* Benzon strain (28). Conclusively, the results from this study confirmed the recessive Cry1Ac-R1 trait conferred by the *ABCC2* mutation, as reported previously (26, 28), regardless of the susceptible *T. ni* strains used to generate the backcross families.

**TABLE 2:** Genotypes of *ABCC2* alleles in the backcross family individuals with and without Cry1Ac selection

Backcross family	Cry1Ac selection	Genotype *ABCC2*^*R*^*ABCC2*^*R*^	Genotype *ABCC2*^*S*^*ABCC2*^*R*^	*P* value^*a*^
TnCry1Ac-R × Cornell → F_1_ × TnCry1Ac-R	0	13	13	1.0
	250 µg/mL	33	0	<0.01
TnCry1Ac-R × Benzon → F_1_ × TnCry1Ac-R	250 µg/mL	51	0	<0.01

^*a*^ *P *values were from chi-square tests with the expected ratio of *ABCC2^R^ABCC2^R^*:* ABCC2^S^ABCC2^R^* = 1:1 in the backcross populations. *ABCC2^R^*: *ABCC2 *allele from the resistant TnCry1Ac-R strain. *ABCC2^S^*: *ABCC2 *allele from the susceptible strains (Cornell strain or Benzon strain).

The results from genotyping the *APN1* alleles were consistent with the expected equal numbers of homozygous *APN1^R^APN1^R^* to heterozygous *APN1^R^APN1^S^* individuals in the two backcross families without Cry1Ac selection (Table 3). However, the ratios of the two *APN1* alleles in the Cry1Ac-selected individuals were drastically different between the two backcross families generated with the two susceptible strains with different genetic backgrounds, indicating differential dominance levels of Cry1Ac-R2 in the two backcross families in response to Cry1Ac at 250 µg/mL (Table 3). The ratio of the two *APN1* genotypes in 16 survivors from the backcross family generated with the Cornell strain was 13 *APN1^R^APN1^R^* to 3 *APN1^R^APN1^S^*, showing selection of the genotype *APN1^R^APN1^R^* over *APN1^R^APN1^S^* under high-dose Cry1Ac treatment (Table 3). In contrast, the ratio of the *APN1* genotypes in 50 survivors from the backcross family generated with the Benzon strain was 26 *APN1^R^APN1^R^* to 24 *APN1^R^APN1^S^*, which is not significantly different from the homozygote-to-heterozygote ratio of 1 to 1 (Table 3). This result indicates that the heterozygotes *APN1^R^APN1^S^* were similarly resistant as the homozygotes *APN1^R^APN1^R^* under *ABCC2^R^ABCC2^R^* in the backcross family generated with Benzon strain.

**TABLE 3:** Genotypes of *APN1* alleles in the backcross family individuals with and without Cry1Ac selection

Backcross family	Cry1Ac selection	Genotype *APN1*^*R*^*APN1*^*R*^	Genotype *APN1*^*R*^*APN1*^*S*^	*P* value^*a*^	Dominance (H)^*b*^
TnCry1Ac-R × Cornell → F_1_ × TnCry1Ac-R	0	11	15	0.43	–
	250 µg/mL	13	3	0.01	0.23
TnCry1Ac-R × Benzon → F_1_ × TnCry1Ac-R	0	15	15	1	–
	250 µg/mL	26	24	0.78	0.92

^*a*^ *P *values were from chi-square tests with the expected ratio of *APN1^R^APN1^R^*:* APN1^R^APN1^S^* = 1:1 in the backcross populations. *APN1^R^*: *APN1 *allele from the resistant TnCry1Ac-R strain. *APN1^S^*: *APN1 *allele from the susceptible strains (Cornell strain or Benzon strain).

^*b*^ – indicates no-toxin treatment controls.

The resistance associated with Cry1Ac-R1 alone without the presence of Cry1Ac-R2 in *T. ni* (genotype of *ABCC2^R^ABCC2^R^ APN1^S^APN1^S^*) is not sufficient for the larvae to survive the Cry1Ac selection at 250 µg/mL (34). In other words, no *APN1^S^APN1^S^* larvae with *ABCC2^R^ABCC2^R^* could survive at 250 µg/mL Cry1Ac. Also, it was confirmed that the backcross families prior to Cry1Ac selection contained approximately equal numbers of *APN1^R^APN1^R^* and *APN1^R^APN1^S^* individuals (Table 3) (48). Therefore, estimation of the dominance of resistance, *h* = (survival of RS − survival of SS)/(survival of RR − survival of SS) (49), can be simplified to *h* = (number of *APN1^R^APN1^S^* survivors)/(number of *APN1^R^APN1^R^* survivors) in this study. The dominance *h* at 250 µg/mL of Cry1Ac in the backcross family generated with the Cornell strain was calculated to be 0.23, showing as an incompletely recessive resistant trait (Table 3). However, the dominance *h* in the backcross family generated with the Benzon strain was 0.92, showing as an incompletely dominant resistant trait (Table 3).

## DISCUSSION

In this study, the second Cry1Ac resistance gene *Cry1Ac-R2* in *T. ni* was mapped to a 675-kb locus on chromosome 9 in the *T. ni* genome. The Cry1Ac resistance evolved in *T. ni* greenhouse populations is shown to be recessive (12, 50), typical of “mode 1”-type resistance to Cry1A toxins commonly observed in Lepidoptera (14). The major Cry1Ac resistance trait Cry1Ac-R1 in *T. ni* has been previously identified to be conferred by a frameshift mutation in *ABCC2*, but it has also been revealed that the high-level resistance to Cry1Ac involves an additional incompletely dominant resistance trait, Cry1Ac-R2, genetically independent of Cry1Ac-R1 (26, 34). As the major resistance trait Cry1Ac-R1 is recessive, previous genetic linkage analyses of Cry1Ac resistance were conducted accordingly based on its recessive resistance, in which homozygous resistant individuals and heterozygous individuals are present in mapping populations constructed to identify the association of recessive resistant alleles (26, 28). In such analyses for recessive resistance, the Cry1Ac-R2 as a secondary and incompletely dominant trait does not distinctively show as an independent resistance trait of the major recessive resistant trait Cry1Ac-R1. To reveal Cry1Ac-R2 as an independent trait requires the trait to be analyzed under the Cry1Ac-R1 (*ABCC2-*knockout) background. Therefore, in this study, the mapping populations with *ABCC2-*knockout were generated by crossing the resistant TnCry1Ac-R strain with an *ABCC2* knockout *T. ni* strain (ABCC2E4-3 strain in this study) and used to analyze the genetic linkage of Cry1Ac-R2 as a dominant trait with markers under the background of *ABCC2* knockout mutation. Using such an experimental approach, we successfully mapped the incompletely dominant secondary resistance locus in the *T. ni* genome.

The Cry1Ac-R2 trait in the *ABCC2* knockout background was previously determined to be incompletely dominant based on the LC_50_s of Cry1Ac in the heterozygous F_1_ *T. ni* larvae (26, 34). In this study, a high dose of Cry1Ac at 250 µg/mL (7 µg/cm^2^) effectively selected the *Cry1Ac-R2* heterozygous survivors by killing the homozygous *Cry1Ac-S2* individuals in the backcross families, allowing for the identification of the association between the Cry1Ac-R2 trait and the molecular markers in the *T. ni* genome. The genetic association analysis by BSA-seq indicated that Cry1Ac-R2 was on chromosome 9 with no associations with the other 30 chromosomes nor with chromosome 0 (*T. ni* genome sequences not been anchored to the 31 specific chromosomes) (Fig. 2). This finding confirmed that Cry1Ac-R2 in *T. ni* is associated with neither *ABCC3* (on chromosome 15) nor the *cadherin* (on chromosome 6), two genes that have been identified to carry mutations, in addition to the *ABCC2* mutation, to confer high-level Cry1Ac resistance in *H. virescens* and *P. xylostella,* respectively (25, 51). Further fine mapping of Cry1Ac-R2 on chromosome 9 by Amp-seq analysis localized Cry1Ac-R2 in a 675-kb region, where the cluster of *APN* genes reside (Fig. 3). The localization of Cry1Ac-R2 in the locus of APN genes is consistent with our previous observations that the absence of 110 kD APN1 is tightly associated with Cry1Ac resistance (26, 46, 47) but is genetically independent of the resistance-conferring mutation in the *ABCC2* gene (34).

This study also discovered that the dominance of Cry1Ac-R2 under a high dose of Cry1Ac could be significantly influenced by the genetic backgrounds of *T. ni* strains (Table 3). Under selection at 250 µg/mL Cry1Ac, the Cry1Ac-R2 trait in the *T. ni* backcross family with the Cornell strain background showed as an incompletely recessive trait (*h* = 0.23), whereas under the same high dose of Cry1Ac, the dominance of Cry1Ac-R2 in the backcross family with the Benzon strain background exhibited as an incompletely dominant trait (*h* = 0.92) (Table 3). This finding satisfactorily explained the seemingly inconsistent previous observations for the association of Cry1Ac resistance with the APN genes using the two different susceptible *T. ni* strains in genetic linkage analyses (28, 46). In previous studies, the *T. ni* Cornell strain was used as the susceptible parent to cross with the near-isogenic resistant TnCry1Ac-R strain to generate the segregating backcross families for the association analysis of midgut APN1 protein and APN1 gene expression with Cry1Ac resistance, and the results showed that the association of lack of APN1 as well as the altered expression level of *APN1* with Cry1Ac resistance appeared to be recessive under selection at a high Cry1Ac dose (500 µg/mL) (46). The *T. ni* Benzon strain has also been used as the susceptible parent to generate the segregating backcross families to analyze the genetic association of *APN* genes with Cry1Ac resistance, and the results showed that both homozygous genotypes of *APN1* and *APN6* from the resistant strain and the heterozygous genotypes with an allele from the susceptible strain survived a high dose of Cry1Ac (28, 46). Therefore, the results from this study and those of previous studies revealed that under a high dose of Cry1Ac, the APN-associated Cry1Ac-R2 trait shows as a recessive trait in the Cornell strain background but shows as a dominant trait in the Benzon strain background (Table 3).

The identification and mapping of the secondary dominant Cry1Ac-resistant trait in *T. ni* from this study indicated that resistance to Cry1Ac in an insect may involve multiple gene mutations conferring dominant or recessive resistant traits, and the dominance of a resistance trait can be influenced by the genetic background of an insect population. Resistance to a Bt toxin in insects commonly appears incompletely recessive, and mapping of the resistance is conducted using segregating backcross populations structured for recessive resistance analysis. However, such genetic linkage analyses designed for recessive resistance traits could overlook dominant secondary resistance traits. The secondary resistance traits may only show a significant resistance effect in the presence of the primary resistance mechanism, and dominant secondary resistant traits will not be detected in mapping experiments for recessive resistance, as the homozygous resistant genotype and heterozygous genotype are not effectively differentiated under a diagnostic toxin dose. Therefore, effective mapping of a secondary resistance trait needs to be conducted in the presence of the primary resistant allele and designed based on its dominance of inheritance, as was conducted in this study for the mapping of Cry1Ac-R2 in *T. ni*. The results from this study showed that the dominance of resistance in an insect can be influenced by the genetic background of the insect population. Although the genetic basis underlying the influence by insect genetic backgrounds remains to be understood, the finding from this study has demonstrated that the dominance of a Bt resistance trait at a given treatment dose may differ greatly in insect populations with different genetic backgrounds, which could complicate research results and data interpretations and impact the efficacy of Bt toxins for insect control and management of Bt-resistance in the field.

In addition to *T. ni*, differential expression of APN genes has also been reported in Cry1Ab-resistant European corn borer, *Ostrinia nubilalis*, and Cry1Ac-resistant diamondback moth, *P. xylostella* (52, 53). Resistance to Cry1Ab in *O. nubilalis* is incompletely dominant, and the resistance has been mapped to a major locus and another minor locus, but both resistance loci are independent of the linkage group of *APN* genes, suggesting that the altered *APN* expression is regulated in *trans* (52, 54). High-level resistance to Cry1Ac in *P. xylostella* is incompletely recessive, and the resistance-associated differential expression of *APN* genes has been reported to be trans-regulated by an MAPK pathway (55), and an insertion mutation in the promoter of the MAP4K4 gene was found to cause the reduced expression of two APN genes in the resistant *P. xylostella* (56). However, the results from this study on *T. ni* showed that the Cry1Ac-R2 trait in *T. ni* is localized on chromosome 9 but has no association with chromosome 15 where MAP4K4 is localized (Fig. 2). The mapping of Cry1Ac-R2 on chromosome 9 is in agreement with our previous observation that the lack of APN1 in Cry1Ac-resistant *T. ni* larvae is genetically not associated with the *ABCC2* gene that is on chromosome 15 in the same linkage group with *MAP4K4* (34).

The molecular mechanisms for the Cry1Ac-R2-associated differential expression of *APN1* and *APN6* in the resistant *T. ni* larvae remain to be understood. Our previous studies of the midgut brush border membrane proteins have biochemically determined that the Cornell strain lacks APN6 and the TnCry1Ac-R2 strain lacks APN1 (34, 46). Binding of Cry1Ac to *T. ni* APN1 can be detected by ligand blot binding analysis upon separation of the midgut proteins by SDS-PAGE, while APN6 does not show as a distinctive specific protein band by SDS-PAGE, and hence its binding to Cry1Ac remains unknown (46). Our recent study on the binding of Cry1Ac to the midgut brush border membranes from *T. ni* has determined that the binding of Cry1Ac to the midgut brush border membrane vesicles (BBMV) from TnCry1Ac-R2 larvae is reduced, compared to the susceptible Cornell strain, but the reduction is not as much as observed from the TnCry1Ac-R1 strain (34). More specifically, the binding affinity of Cry1Ac to the midgut BBMV from TnCry1Ac-R2 is reduced, but the midgut binding site density remains unchanged, compared to the Cornell strain. This is in contrast to both reduced binding affinity and reduced binding site density of Cry1Ac to the midgut of TnCry1Ac-R1 (34). Understanding how Cry1Ac-R1 and Cry1Ac-R2 interact to lead to increased resistance to Cry1Ac in *T. ni* may provide new insights into the molecular mode of action of Bt toxins and requires further investigation.

## Data Availability

Raw sequencing reads have been deposited into the NCBI Sequence Read Archive (SRA) under accession no. PRJNA1107834.
